# Offline Persistence of Memory-Related Cerebral Activity during Active Wakefulness

**DOI:** 10.1371/journal.pbio.0040100

**Published:** 2006-03-28

**Authors:** Philippe Peigneux, Pierre Orban, Evelyne Balteau, Christian Degueldre, André Luxen, Steven Laureys, Pierre Maquet

**Affiliations:** **1**Cyclotron Research Center, University of Liège, Liège, Belgium; **2**Neurology Department, Centre Hospitalier Universitaire (CHU), University of Liège, Liège, Belgium; MITUnited States of America

## Abstract

Much remains to be discovered about the fate of recent memories in the human brain. Several studies have reported the reactivation of learning-related cerebral activity during post-training sleep, suggesting that sleep plays a role in the offline processing and consolidation of memory. However, little is known about how new information is maintained and processed during post-training wakefulness before sleep, while the brain is actively engaged in other cognitive activities. We show, using functional magnetic resonance imaging, that brain activity elicited during a new learning episode modulates brain responses to an unrelated cognitive task, during the waking period following the end of training. This post-training activity evolves in learning-related cerebral structures, in which functional connections with other brain regions are gradually established or reinforced. It also correlates with behavioral performance. These processes follow a different time course for hippocampus-dependent and hippocampus-independent memories. Our experimental approach allowed the characterization of the offline evolution of the cerebral correlates of recent memories, without the confounding effect of concurrent practice of the learned material. Results indicate that the human brain has already extensively processed recent memories during the first hours of post-training wakefulness, even when simultaneously coping with unrelated cognitive demands.

## Abstract 2

A human fMRI study showing how learning selectively modulates brain activity during wakefulness, providing novel evidence that "offline" activation patterns differ according to the preceding memory task and memory performance.

## Introduction

Human [ 1– 4] and animal [ 5– 9] studies have revealed experience-dependent reactivations of regional cerebral activity during post-training sleep, in brain areas previously engaged in learning during wakefulness. Furthermore, in humans, post-training reactivations in hippocampal ensembles have been found to correlate with overnight improvement in performance in a spatial navigation task [ 2]. Likewise, local increases in slow-wave activity during sleep after learning correlate with improved performance in a motor adaptation task in the post-sleep period [ 4]. Experience-dependent reactivations of cerebral activity are hypothesized to reflect the *offline* processing of recent memories during sleep, which eventually leads to the plastic changes underlying memory consolidation and a subsequent improvement in performance [ 10, 11]. These and other studies (for example [ 12– 14]) have emphasized a prominent role for brain activity during sleep in the offline processes of memory consolidation, which suggests that memories are strengthened and/or restructured mostly during post-training sleep, rather than during immediate post-training wakefulness. From this point of view, recent memories should be maintained in a relatively unaltered form in the waking brain during the period that follows the end of learning but that precedes the first post-training sleep period.

However, sleep probably allows but a few steps in the succession of offline transformations that occur between the initial encoding of a new piece of information and its final incorporation into long-term memory stores. For example, it has been hypothesized that memories are stabilized (i.e. become resistant to interference) during wakefulness and are then consolidated/enhanced during sleep [ 15]. However, an absolute partition of offline memory operations between vigilance states is debatable [ 16]. Other models of memory formation propose that part of the post-training consolidation process takes place during wakefulness in the offline periods of behavioral inactivity that follow the acquisition of new material [ 17, 18]. This suggests that memories reactivated and strengthened during post-training sleep are not only maintained during initial post-training wakefulness, but are also likely to be extensively processed during this period of time. Congruent evidence has arisen from multiple cell recordings studies in rodents [ 5, 8] and in non-human primates [ 19] that shows a coordinated reactivation of practice-related neuronal ensembles immediately following exposure to a new task in the waking period preceding sleep. However, these electrophysiological activities have been found to persist during a restricted period of post-training time only, up to ±15 min [ 5, 19], which may suggest a limited role for these neuronal oscillations in the maintenance of new information in the brain system during wakefulness. In addition, their functional significance remains to be proven, as changes in performance levels between learning and post-reactivation behavioral sessions were not examined in these studies. It is therefore unclear whether the post-training persistence of electrophysiological activity during a limited amount of post-training wakefulness supports the initial steps of memory consolidation, or whether this is merely a neurophysiological consequence of the intense activation of learning-related neuronal ensembles during prior practice.

A further issue, to our knowledge not commonly tackled by cognitive neuroscientists, is the fact that periods of wakefulness occurring immediately after the acquisition of new memories are usually filled with a wide variety of *active* cognitive processes, rather than with behavioral inactivity. Therefore, one might wonder how the human brain faces the challenge of simultaneously processing and/or maintaining recently acquired memories for extended periods of time, for some hours, while successfully coping with unrelated cognitive demands. One hypothesis is that exposure to specific events subsequently modulates brain responses to other cognitive tasks performed during the waking period that immediately follows. In line with this proposal, it is known that exposure to environmental factors, such as bright light, enhances regional cerebral activity in humans during an auditory attentional task performed in darkness immediately after the lit period [ 20]. Conversely, spontaneous ongoing cerebral activity is known to modify profoundly evoked responses to external stimuli in a cat's visual cortex [ 21]. These data thus indicate that ongoing brain activity is not only affected by currently occurring stimuli, but also by the context set by prior inputs. In the framework of the acquisition of new information in a learning task, post-training modulation of ongoing cerebral activity would therefore allow the brain to keep an imprint of recently acquired memories while engaged in unrelated activities.

In the present study, we aimed at characterizing the cerebral correlates of the offline maintenance of recently acquired memories during active wakefulness in man, after training has ended and before the intervention of sleep-related consolidation processes. As stated above, we hypothesized that the acquisition of new information during the learning task would modulate the brain responses to an unrelated probe task performed during the immediately subsequent waking period. However, demonstrating a change in brain response to the probe task after learning is not sufficient to determine whether the modulation actually reflects the persistence of learning-related activity during post-training wakefulness, or whether it is merely a non-specific outcome of extensive stimulation during the training session. Therefore, we compared post-training modulation of brain activity after two learning tasks representative of the main memory systems in influential classifications of memory [ 22, 23]. These memory systems are, firstly, the declarative/spatial memory system, which is thought to be crucially hippocampus-dependent, and secondly, the non-declarative procedural memory system, whose integrity is not primarily dependent on the hippocampus (i.e. it is hippocampus-independent) but rather relies on a set of cortical and subcortical regions including motor and premotor areas, striatum and cerebellum [ 24]. The spatial memory task consisted of place learning in a virtual 3D town [ 25], while the procedural memory task was a multiple choice serial reaction time (SRT) task [ 26], a paradigm of motor sequence learning (see details in the Materials and Methods section). These spatial and procedural memory tasks were selected because they have been shown to induce post-training cerebral activity in learning-related regions during, respectively, slow-wave sleep [ 2] and rapid eye movement sleep [ 1, 3]. Likewise here, we hypothesized that post-training modulation of brain activity during active wakefulness would occur in brain areas specifically associated with the learning type, reflecting the offline maintenance of newly acquired information. It is worth emphasizing that this original approach presents the unique advantage of allowing detection of the post-training evolution of learning-related regional brain activity during wakefulness, uncontaminated by the actual practice of the learning task.

**Figure 1:**
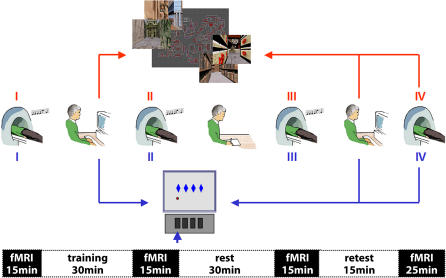
Experimental Design All participants underwent four fMRI scanning sessions (I–IV) within a half-day. In scanning session (I), they performed an auditory oddball task during which they mentally counted the number of deviant tones interspersed in a flow of repeated tones. Participants were then trained during 30 min outside of the scanner (training), either to the spatial memory navigation task (red path), or to the procedural memory SRT task (blue path). Immediately after the end of the training session, they were scanned again (II) while performing the auditory oddball task. They were then allowed a further 30-min break outside of the scanner without any further practice (rest). They were scanned once again (III) while performing the auditory oddball task. Afterwards, participants' memory of the learned task was tested outside of the scanner (retest). Finally, participants underwent a fourth fMRI session (IV), during which they explored virtual environments (red path) or practiced motor sequences in the SRT task (blue path), to determine the set of brain areas associated with task practice. The procedure was repeated 2 wk later using the other learning task.

Our experimental design was as follows. Fifteen healthy volunteers were scanned using event-related functional magnetic resonance imaging (fMRI) while exposed to a probe auditory oddball task at three different sessions in a half-day ( Figure 1). In the auditory oddball, participants were requested to mentally count the number of deviant sounds that occurred in a monotonous flow of repeated tones. Cerebral response to the deviant auditory events was the dependent measure of brain activity at each probe session. The first and second scanning sessions were performed respectively immediately *before* and *after* an episode either of spatial or procedural learning, carried out for 30 min outside the scanner. In order to demonstrate enduring learning-related brain activity immediately after the end of practice, we looked for changes in regional cerebral activity during the post-learning versus the pre-learning (i.e. the baseline) fMRI session. In addition, a third oddball session was conducted after another 30-min break, during which volunteers did not practice the learning task again. This supplementary rest interval allowed us to test for the temporal persistence of post-training cerebral activity up to ±45 min (i.e. the 30-min break plus the time spent in the scanner during the second oddball session) after the end of learning, by assessing changes in cerebral activity from the second to the third fMRI probe session. Afterwards, participants were retested on the learning task outside the scanner, in the same condition as during the initial learning task, in order to measure changes in behavioral performance levels. Finally, they underwent a fourth block-design fMRI session, during which they performed either on the spatial or on the procedural task used for learning, in order to identify the set of brain areas associated with task practice. Two weeks later, the same participants were scanned again under the same protocol but using the other learning task, at the same time of day to avoid any circadian confound. Using this within-subject strategy, post-training changes in regional brain activity specifically related to the spatial memory task could be controlled for post-training activity modifications related to the motor procedural task, and vice-versa.

In summary, this unique experimental design allowed the characterization during active wakefulness of (a) the offline modulation of regional brain responses to the probe task by recent learning in the human brain, (b) the specificity of this modulation to the type of prior learning (i.e. spatial versus procedural), and (c) the evolution of these learning-related modulations at two different post-training time intervals, immediately and 45 min after training had ended.

## Results

### Behavioral Performance

Detailed behavioral results are reported in Protocol S1. Only essential information is provided here.

In the probe auditory oddball task, counting accuracy was 99% on average (range 85–100%) and did not evolve across oddball sessions, F(2, 32) = 1.49, *p* > 0.23, nor did it do so between the two acquisition days, F(1, 16) = 0.21, *p* > 0.65. This suggests that participants remained adequately focused on the probe task all through the experiment.

In the spatial learning task, participants were administered five 90-s tests of place retrieval at the end of learning in the virtual town (between fMRI Sessions I and II) and at retest (after fMRI Session III). Mean distance left to destination at the end of the 90-s period was shorter at retest (21.7 ± 12.3 distance [arbitrary] units ± standard deviation) than immediately after learning (27.1 ± 12.5 units; t[ 1, 14] = 2.10, *p* = .05; Figure S1). However, one cannot rule out the possibility that the five tests performed at the end of the learning session provided participants with feedback that partially contributed to the limited improvement in performance after the 1-h interval. This change in performance was moreover far behind previously reported levels of overnight improvement using the same material [ 2]. Therefore, following a conservative interpretation, these results indicate spatial memory maintenance in the navigation task over a 1-h interval.

In the SRT task, 30 blocks of SRT practice (L1–L30) each containing eight repetitions of a 12-element sequence of locations were administered during learning (between fMRI Sessions I and II), then nine blocks (T1–T9) during retest (after fMRI Session III). In order to assess the extent to which participants learned the trained sequence, another sequence was presented during blocks L28, T2, and T8. Increased reaction time (RT) from the learned to the unlearned sequence was significant both within Learning (L28 versus L27) and Testing (T2 versus T1) sessions ( *p* < 0.0005; Figure S2), indicating that participants had acquired specific knowledge about the sequential regularities characteristic of the trained sequence. A two-way analysis of variance (ANOVA) using blocks L27–L28 and T1–T2 (interaction effect F[ 1, 14] = 12,746, *p* < 0.005) indicated that RTs improved from the Learning to the Testing phase for the untrained sequence (518 ± 58 versus 458 ± 53 ms, *p* < 0.005, post-hoc HSD Tukey test), but not for the learned sequence (309 ± 49 versus 298 ± 46 ms, *p* > 0.5). Data inspection indicated a ceiling effect in RT performance for the learned sequence (see Supporting Information). These results suggest that knowledge of the sequential regularities remained stable between learning and retest sessions over the 1-h interval. Since no explicit memory test was administered at the end of the SRT experimental session, we cannot determine here the extent to which participants became aware of the sequential pattern of the learned sequence. Nevertheless, it has been demonstrated that practice using this same material with a response-stimulus interval of 0 ms, which we used here, mostly promotes implicit knowledge of the regularities of the sequence in the deterministic SRT task [ 27, 28].

### Learning Modulates Regional Cerebral Activity during Post-Training Wakefulness

In keeping with our hypothesis, regional blood oxygen level-dependent (BOLD) response in practice-related areas was modified in a task-specific manner by prior learning. Tables S1 and S2 provide a list of brain areas in which post-training activity increased or decreased immediately and 45 min after practice, computed separately within the context of spatial learning ( Table S1) or procedural learning ( Table S2). These main effects were used to validate the interpretation of Session by Learning Task interaction effects reported below.

**Figure 2:**
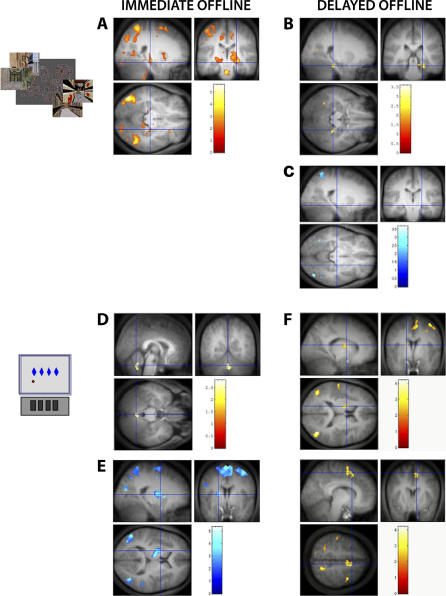
Task-Specific Modulation of Regional Brain Responses by Prior Learning Spatial learning-related offline activity: (A) Higher brain responses after spatial than after procedural learning in Session II (versus I). Blue cross hair on hippocampus (26 −24 −8 mm) activation superimposed on participants' average anatomical T1-weighted MRI image. (B) Higher brain responses in the parahippocampal gyrus (26 −32 −18 mm) after a further 30-min break during Session III (versus II). (C) Co-occurring decreased brain responses in the hippocampus (22 −22 −10 mm, blue cross hair) during Session III (versus II), more after spatial than after procedural learning. Procedural learning-related offline activity: (D) Higher brain response in the medial cerebellum (2 −60 −28 mm) after procedural than after spatial learning in Session II (versus I). (E) Co-occurring decreased brain responses in the putamen (−20 2 10 mm, blue cross hair), lateral cerebellum, SMA, and other neocortical areas during Session II (versus I), more after procedural than after spatial learning. (F) Higher brain response after a further 30-min break during Session III (versus II) in the caudate nucleus (top: −16 0 16 mm) and the SMA (bottom: 10 2 56 mm). Color bars indicate the magnitude of the effect size, in the yellow range for increased post-training brain response, and in the blue range for decreased post-training brain response.

**Figure 3:**
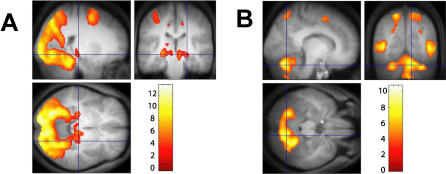
Practice-Related Activations (A) Brain activity during exploration of the virtual environment (Session IV). Cross hair shows hippocampus activation (22 −26 −6 mm, *p* ^corr^ < 0.005) superimposed on participants' average anatomical T1-weighted MRI image. Color bars indicate magnitude of effect size. (B) Brain activity during practice of the procedural serial RT task (Session IV). Cross hair shows cerebellum activation (12 −74 −22 mm, *p* ^corr^ < 0.05).

Immediately after spatial learning, brain responses to the probe task ( Figure 2; Table 1) were significantly larger than in the pre-training session (i.e. Session II versus I), and more so than after procedural learning, bilaterally in the hippocampus and in the parahippocampal gyrus ( *p* ^svc^ < 0.05; Figure 2A) at coordinates activated during task practice ( Figure 3) and previously associated with spatial navigation and place finding in virtual environments (e.g. [ 25]). Similar increased responses were found in a distributed set of cortical and subcortical areas ( *p* ^corr^ < 0.05; Table 1) including the retrosplenial cortex, the thalamus, the cuneus, the superior parietal lobule, and the superior frontal gyrus. We found no area in which activity decreased immediately after spatial training (Session I versus II; Table 1). Thirty min later (Session III versus II; Table 1), brain activity further increased in the left parahippocampal gyrus after spatial learning ( *p* ^svc^ < 0.05; Figure 2B). At the same time, post-training activity decreased from Session II to III in the hippocampus and in another portion of the parahippocampal gyrus located at the junction with the lingual gyrus ( *p* ^svc^ < 0.05; Figure 2C), and in the middle temporal and medial frontal gyri ( *p* ^corr^ < 0.05), more so after spatial than after procedural learning ( Table 1). Nonetheless, delayed post-training activations remained significantly higher in the hippocampus, the parahippocampal gyrus and the medial frontal gyrus during delayed post-training Session III than during pre-training Session I ( *p* ^svc^ < 0.05). This indicates that increases in post-training activity are preserved in these areas during a 1-h interval. We found no area in which activity conversely decreased immediately after spatial training then increased later on. These results indicate that post-training activity in navigation-related areas ( Figure 3A), and especially in the hippocampal region, increases immediately after spatial learning then persists over time, except in the left parahippocampal area, in which a further increase is subsequently observed.

**Table 1:**
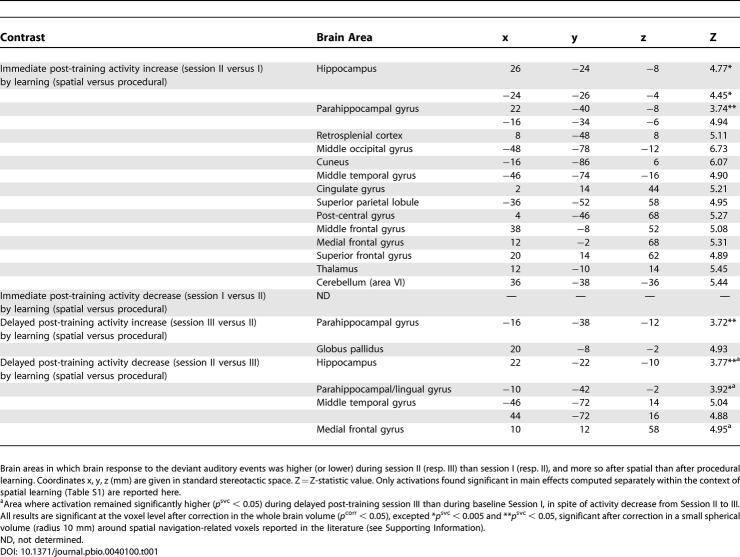
Offline Activity after Spatial Learning

The converse Session by Learning Task interaction analyses tested whether brain responses to the probe task were modified by prior procedural learning ( Table 2) in regions activated during SRT practice ( Figure 3B), and more so than by spatial learning. Immediately after procedural learning (Session II versus I), we found increased brain response medially in the cerebellum ( *p* ^svc^ < 0.05; Table 2), near to the fastigial nucleus and the secondary fissure ( Figure 2D). Concurrently, there was an immediate decreased response (Session I versus II; Figure 2E) in the putamen, the thalamus and the right lateral cerebellum (Crus II), as well as in a set of visuomotor-related neocortical areas including the cuneus and the precuneus, the post-central, middle temporal and middle frontal gyri, the supplementary motor area (SMA) and the superior parietal lobule ( *p* ^corr^ < 0.05; Table 2). Afterwards, a delayed increase (Session III versus II) occurred in the caudate nucleus and in the SMA ( *p* ^svc^ < 0.05; Figure 2F), around coordinates activated during task practice ( Figure 3B) and previously associated with motor sequence learning (e.g. [ 29, 30]). Similarly, delayed increases were found in a set of visuomotor-related neocortical areas, including pre-SMA, precuneus, post-central gyrus, and middle temporal and frontal gyri ( *p* ^corr^ < 0.05; Table 2). Importantly, activations in the caudate nucleus, SMA, pre-SMA and middle frontal and post-central gyri were significantly higher during the delayed post-training Session III than during the pre-training Session I ( *p* ^svc^ < 0.05). This indicates that the delayed increase from Session II to III was not merely the recovery of pre-training levels of activity after the immediate post-training decrease during Session II. Finally, a delayed decrease of activity was found in the left lateral cerebellum only (Session II versus III; *p* ^corr^ < 0.05; Table 2). These results show that the immediate post-training time period is mostly characterized by a decrease in brain response in a set of cortical and subcortical regions involved in task performance, co-occurring with an increase in activity in the medial part of the cerebellum. The initial decrease in post-training activity is then followed by a delayed increase, which exceeds pre-training levels in learning-related areas. In the basal ganglia, in particular, we found an initial decrease in activity in the putamen, followed by a subsequent increase in activity in the caudate nucleus, representing a delayed stage in the offline activity that takes place after procedural learning has ended (see also Figure S3).

**Table 2:**
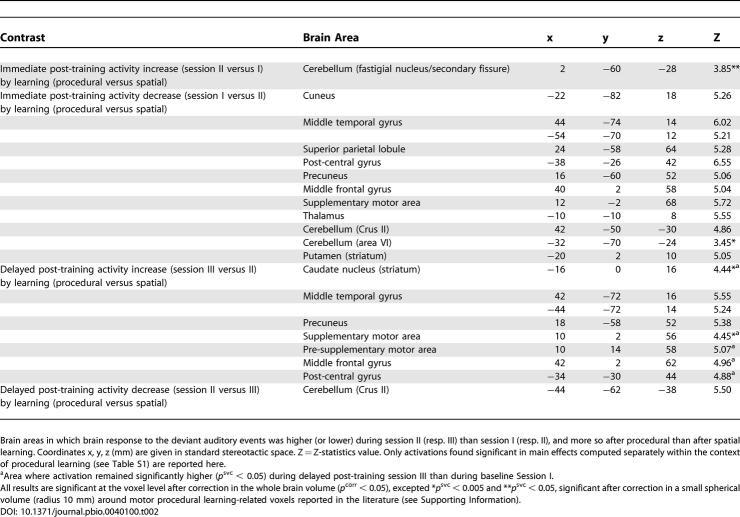
Offline Activity after Procedural Learning

### Changes in Functional Integration in the Offline Period after Learning

**Figure 4:**
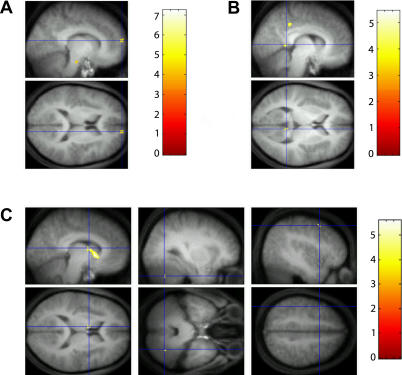
Offline Modulation of Cerebral Connectivity during Post-Training Wakefulness Offline spatial learning-related connectivity: (A) Tighter coupling during Session II than during Session I between hippocampus (at coordinate 26 −24 −8 mm) and superior frontal gyrus activity (cross hair at [12 66 16 mm] Z = 3.87; *p* ^svc(10mm)^ < 0.05), superimposed on participants' average anatomical T1-weighted MRI image. Color bars indicate magnitude of effect size. (B) Delayed tighter coupling during Session III than during Session I between hippocampus (26 −24 −8 mm) and retrosplenial cortex (cross hair at [8 −48 8 mm]), Z = 3.42, *p* ^svc(10mm)^ < 0.05). (C) Offline procedural learning-related connectivity: Delayed enhancement in coupling during Session III as compared to Session I, between cerebellum activity (at coordinate 2 −60 28 mm) and activity in the caudate nucleus (left panel: [−8 2 14 mm] [cross hair], and [−18 −14 24 mm], Z = 3.99 and 3.71, *p* ^svc(10mm)^ < 0.05), the putamen ([20 2 −4 mm], data not shown, Z = 3.56, *p* ^svc(10mm)^ < 0.05), the lateral cerebellum (middle panel: cross hair at [32 −66 −36 mm], Z = 3.33, *p* ^svc(10mm)^ < 0.05), and the dorsal premotor cortex (right panel: cross hair at [−44 10 54 mm], Z = 3.16, trend *p* ^svc(10mm)^ = 0.07).

Psychophysiological interaction analyses ( Figure 4) tested the complementary hypothesis that those areas showing offline, learning-dependent, modulation of their activity would gradually establish or reinforce functional connections with other brain regions. Results showed that BOLD response in the right hippocampus (coordinates 26 −24 −8 mm) was more tightly coupled immediately after spatial learning than before (Session II versus I) with BOLD response in the superior frontal gyrus ( *p* ^svc^ < 0.05; Figure 4A), an area known to be activated during successful strategic route finding in a virtual town [ 25]. Functional connectivity further increased during Session III (versus I) between the hippocampus and the retrosplenial area ( *p* ^svc^ < 0.05; Figure 4B), another region known for its involvement in human navigation [ 31]. Conversely, activity in the medial cerebellum (2 −60 −28 mm) was more tightly coupled after a 45-min delay ( *p* ^svc^ < 0.05; Session III versus I) following the end of motor procedural learning, but not immediately after practice (Session II versus I), with activity in the caudate nucleus ( Figure 4C), a structure associated with both successful learning of complex motor sequences [ 29] and their offline processing during post-training REM sleep [ 1]. Tighter coupling of cerebral activity was also found in the cerebellum laterally in the lobus semi-lunaris superior (Crus I), the putamen, and the dorsal premotor cortex ( Figure 4C), all areas implicated in the delayed processing of learned sequences [ 24, 30].

These results suggest that task-dependent and regionally-specific changes in functional integration progressively take place during the post-training waking period either after spatial or after procedural learning, but following a different time course. After spatial learning, hippocampal functional connectivity progressively involves frontal then retrosplenial cortical regions. After procedural learning, a delayed maturation of cerebello-frontal and cerebello-striatal connectivity occurs offline at some point after the end of immediate post-training Session II, from 15–45 min after the training phase.

### Behavioral Correlates of the Offline Processing of Memories during Wakefulness

There is a possibility that these results represent idling activities without any behavioral impact. In order to assess the functional significance of these phenomena in memory processing, we tested whether offline modifications of neuronal activity relate to the maintenance of the recently acquired memories, as assessed behaviorally. As shown above, average group performance stabilized across the 1-h interval between learning and retest phases both in spatial navigation and motor sequence learning conditions (see also Supporting Information).

**Figure 5:**
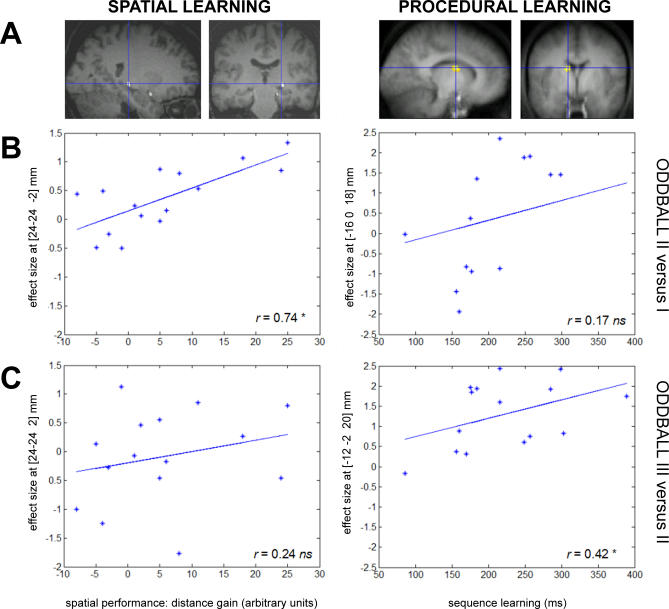
Post-Training Modulation of Neuronal Activity and Behavioral Performance (A) Activations are superimposed on one participant's T1-weighted normalized MRI image. Left side: Plots of the correlation between changes in spatial performance (distance left to target in learning minus test sessions) and brain response during intervening oddball Session II (versus I; [B]) in the hippocampus ([24 −24 −2 mm], Z = 3.75, *p* ^svc(10mm)^ < 0.05) around an a priori location [26 −24 −8 mm]). Each point represents one participant. Part C shows the non-significant correlation ( *p* > 0.8) at the same location during Session III (versus II). Right side: Plots of the correlation between individual levels of sequence knowledge (RT for novel minus learned sequence) at the end of the Learning phase and brain response during (B) Session II (versus I), showing the non-significant correlation in the left caudate nucleus, and (C) intervening oddball Session III (versus II[C]) in the same location ([−12 −2 20 mm], Z = 4.48, *p* ^svc^ < 0.005). *r* = correlation coefficient.

For spatial memories, a positive correlation was found between individual changes in spatial performance (from learning to retest behavioral sessions) and right hippocampal response during the intervening immediate post-spatial training Session II (versus I; Pearson correlation coefficient *r* = 0.74, *p* ^svc^ < 0.05; Figure 5). The correlation was no longer significant during Session III (versus II). This finding is reminiscent of a previously reported correlation between overnight performance improvement to the same task and hippocampal activity during post-training slow-wave sleep [ 2]. For procedural sequence learning, a similar analysis failed to reveal a significant correlation between changes in levels of sequence knowledge (i.e. the change in RT difference between learned and novel sequences, from the learning to the retest session) and post-procedural training responses in learning-related areas. However, we found that response in the left caudate nucleus during the delayed Session III (versus II) was proportional to the level of sequence knowledge at the end of the learning behavioral session ( *r* = 0.42, *p* ^svc^ < 0.05; Figure 5), as well as at retest ( *r* = 0.29, *p* ^svc^ < 0.05). No significant correlation was found during Session II (versus I). The correlation between learning levels of performance and delayed post-procedural training activity during active wakefulness is reminiscent of our previous finding that levels of sequence learning measured at the end of training correlate with the amplitude of offline neuronal reactivation during post-training REM sleep [ 1].

The functional relationship between behavioral performance and brain response in learning-related structures at specific time intervals (i.e. Session II or III) during the intervening waking period further suggests that these neural activities are involved in the processing of recently acquired information.

## Discussion

Neuroimaging studies have usually assessed the temporal and spatial evolution of the neuronal correlates of recent memories by scanning participants during the practice of a learning task, i.e. online, repeatedly after variable resting intervals. Here we characterized the offline evolution of the cerebral correlates of these recent memories, without the confounding effect of any concurrent practice of the learned material. Hence this paradigm reveals the neuronal activity underlying the maintenance of latent memories. Furthermore, we show that post-learning persistence and early reorganization of neuronal activity during wakefulness is a common feature both for hippocampus-independent (motor procedural) and hippocampus-dependent (spatial) memories, but with different time courses.

In the initial stages of motor sequence learning, cortico-cerebellar circuits are preferentially activated [ 32], whereas after extended practice, delayed recall involves cortico-striatal networks [ 32– 34]. Our results suggest that the cortico-cerebellar and cortico-striatal networks interact very early on during post-training wakefulness, in line with evidence for a pathway enabling the output stage of cerebellar processing to have a direct influence on the input stage of basal ganglia processing [ 35]. We also found that post-procedural training activity is mostly characterized by an immediate decrease in brain response, followed by heightened activity in the striatum and motor-related neocortical areas. Decreased activity in the basal ganglia [ 36, 37], pre-SMA, and frontal cortex [ 37] has been reported to occur during the early phase of learning a sequence of movements, whereas increased striatal activity has been found at an advanced phase of motor sequence learning [ 32, 38]. In addition, early and advanced sequence learning appear to engage separate entities within the basal ganglia [ 39, 40]. The temporal and spatial dynamic of these activities during post-training wakefulness may contribute to the heralding of changes in functional segregation observed during practice at a later date [ 24, 30]. Together with behavioral data [ 41], these results suggest multiple shifts in latent representations of motor experience after the acquisition of skilled performance.

It is known that partially overlapping hippocampal and cortical regions are involved in both retrieval and encoding of declarative and spatial memories [ 42]. This makes it difficult to investigate the cerebral correlates of the evolution of spatial memories during repeated practice of a task, since online processing of the stimuli will always involve both encoding and retrieval components. Nonetheless, both rodent and human studies support the hypothesis that memories are rapidly encoded in hippocampal networks, but are only progressively transferred to cortical networks so that their final repository lies in the neocortex [ 43] (but see [ 44]), such as the retrosplenial and cingulate cortices [ 18]. Accordingly, retrieval-related activity in the hippocampus does not diminish in a recognition memory task performed immediately, 1 d or 1 wk after learning [ 45], and has even been found to increase in the hippocampal-neocortical network after 1 mo [ 46, 47], which suggests that hippocampal disengagement is a long-term process. Our present data revealed sustained offline activity in the hippocampal formation and a large set of navigation-related cortical and subcortical areas. This activity takes place immediately after spatial learning and persists over a 1-h interval. This result is in keeping with the rapid development of stable patterns of neuronal responses in the rat hippocampus following exposure to a novel environment [ 48, 49], as well as with the instantiation of a neocortical imprint for these spatial memories. Further studies need to investigate whether offline hippocampal post-training activity still persists or fades away when spatial memories become enduringly stored at the neocortical level.

To the best of our knowledge, persistence and spatial reorganization of cerebral activity during post-training wakefulness have been reported at different levels, but have never been directly related to changes in behavior, nor have they been assessed in the context of ongoing but unrelated cognitive demands (i.e., the probe task). In rodents, the induction of long-term potentiation in the dentate gyrus of the hippocampus has been shown to lead to the upregulation of zif-268 gene expression locally at the stimulation site after 30 min and in surrounding brain areas after 3 h of sustained wakefulness [ 50]. Also, stimulation leading to long-term potentiation in the hippocampus can induce sharp wave-ripple complexes [ 51], thought to be critical for the stabilization of memory traces in the cortex and known to occur spontaneously during behavioral immobility and slow-wave sleep [ 17]. At the microscopic systems level, the distribution of pairwise correlations in neuronal firing rates within CA1 is maintained during offline periods of quiet wakefulness [ 8]. Likewise, spatio-temporal patterns of neuronal activity are repeated in the hippocampus, the putamen, and the thalamus for up to 48 h after the exploration of a novel environment [ 6]. In the macaque, simultaneous multi-unit recordings in several neocortical sites have revealed continued coactivation patterns of cell activity during the behaviorally inactive period (±10 min) following the practice of a series of reaching tasks [ 19]. It should be kept in mind, however, that the hemodynamic changes estimated by BOLD responses are likely to reflect the energetically expensive synaptic activity related to the local field potential signals, i.e. the input and local processing in a brain area, more than the neuronal spike rate per se [ 52]. This may explain why we found traces of continued brain activity during post-training wakefulness up to 1 h after learning, whereas hippocampal and neocortical electrophysiological activations seem to vanish after about 15 min [ 5, 8, 19]. At the systems level, a time-dependent increase in [ _14_C]2-deoxyglucose uptake occurs at a slower time scale in rodents during the offline rest period following operant conditioning, first in subcortical and limbic areas (thalamus, hippocampus) and, more than 3 h later, in neocortical regions [ 53]. In humans, functional connectivity in resting-state networks is affected by immediate prior cognitive state [ 54]. We also found that post-training changes in regional brain activity relate to performance, suggesting their functional implication in the processing and maintenance of recent memories. Although the cellular correlates of the post-training changes in regional brain responses are not yet known in humans, both increased and decreased responsiveness of neuronal ensembles persist immediately after training and spread progressively to distant brain areas. Early modifications in neural responsiveness during offline memory processing possibly rely on molecular processes similar to those characterized in animals, such as long-term potentiation [ 55], molecular cascades triggered by early transcription [ 56] or wiring plasticity [ 57].

Finally, the present study demonstrates learning-dependent changes in spontaneous regional brain activity during post-training wakefulness, similar to learning-dependent changes during post-training sleep [ 1– 4], both for hippocampus-dependent and hippocampus-independent memories. Though these spontaneous offline activities may appear phenomenally similar, it is worth remembering that sleep and wakefulness are strikingly different vigilance states characterized by specific neuronal firing patterns, neuromodulatory context and gene expression [ 58]. The question remains unanswered as to how these parameters affect the functional status of the offline persistence of post-training cerebral activity for the processing and consolidation of recent memories during sleep and wakefulness. The present results suggest that post-training changes in regional cerebral activity during the first hours of post-training wakefulness are an integral part of the processing and maintenance of recent memories in the human brain, even when it is currently coping with unrelated cognitive demands.

## Materials and Methods

More detailed descriptions of the learning tasks and fMRI analysis methods are available in Protocol S1. Only essential information is provided here.

### Participants

Fifteen right-handed healthy volunteers (nine males and six females; age range 20–29 y) gave their written informed consent to take part in this study approved by the Ethics Committee of the University of Liège. None of the participants declared any neurological or psychiatric disease history, nor were they using any centrally acting medication. They were explicitly required not to consume drugs or alcohol and to restrict their caffeine intake for 24 h prior to each experimental day. Participants were paid for their participation in the experiment. Sleep quality (see Supporting Information) was similar between the nights preceding each half-day of testing, as well as between the nights preceding the procedural versus the spatial learning task in the protocol (all *p* > 0.11; Wilcoxon tests).

### Behavioral tasks

In the auditory oddball task, participants were requested to mentally count the number of deviant tones (±30 events) that occurred in a monotonous flow of repeated tones (±270 events), while keeping their eyes centered on a fixation cross. They had to report their count after the end of scanning. Pure tones of 300 and 400 Hz (duration 600 ms; inter-stimulus interval 1,000 ms) were presented using magnetic resonance imaging (MRI)-compatible electrodynamic earmuff headphones with gradient noise suppression (MR confon GmbH, Magdeburg, Germany). The auditory oddball was chosen as the probe task because it does not lead to any learning by itself, and brain responses are highly reproducible over time [ 59]. This makes it easier to detect modulations of regional brain activity (i.e. changes in BOLD response) related to prior learning experience. Each oddball fMRI session lasted ±15 min including participants' installation in the scanner.

For the spatial navigation task, the virtual environment adapted from [ 2] was created and presented using a commercially available computer game (Duke Nukem 3D, 3D Realms Entertainment, Apogee Software Ltd., Garland, Texas, United States). Participants had a color 3D, first-person, view from inside an enriched environment, in which they navigated at constant speed using arrow keys. In the walking area, three target objects were identified by a rotating medallion (e.g. the Buddha statue, Figure 1). During learning (between fMRI Sessions I and II), participants were instructed to learn the topography of the town during three exploration periods of 7.5 min. During tests or route finding (at the end of learning and after fMRI Session III), participants were designated a starting location and instructed to reach a remote object in no more than 90 s. After this time had elapsed, the distance remaining between the participant's actual location and his/her final destination was computed using the shortest possible path (arbitrary units) and used as a quantitative estimate of topographical knowledge (i.e. the shorter the remaining distance to the destination, the better the performance).

In the SRT task, participants faced a screen where four permanent position markers were displayed horizontally above four spatially compatible response keys. A single SRT block consisted of 96 successive trials. On each trial, a black dot appeared 2 cm below one of the position markers, and the task consisted of pressing as fast and as accurately as possible with the right hand on the corresponding key. Response-stimulus interval was 0 ms; errors were indicated by a visual display. Not indicated to participants, each block contained eight repetitions of one out of two 12-element sequences of locations. Thirty blocks of SRT practice (L1–L30) were administered during learning (between fMRI Sessions I and II) and nine blocks (T1–T9) during the retest (after fMRI Session III) using the same sequence, except for blocks L28, T2, and T8, during which the other sequence was presented. Individual levels of sequence knowledge improvement were estimated based on the difference from learning to retest sessions between RTs for the trained versus the novel sequences ([L29 minus L28] *minus* [T2 minus T1]; i.e. positive values meant improvement).

The order of presentation of learning tasks over the 2 d was randomized across participants.

### fMRI acquisition

Data were acquired on a 3 Tesla head-only MRI scanner (Allegra, Siemens Medical Systems, Erlangen, Germany) using a T2* sensitive gradient echo (EPI) sequence (TR 2,130 ms, TE 40 ms, FA 90°, matrix size 64 × 64 × 32; voxel size: 3.4 × 3.4 × 3 mm ^3^). Thirty-two contiguous 3-mm thick transverse slices were acquired, covering the whole brain. Anatomical images were obtained at the end of one of the two half-days by using a T1-weigthed 3D MP-RAGE sequence (TR 1,960 ms, TE 4.43 ms, TI 1,100 ms, FOV 230 × 173 cm ^2^, matrix size 256 × 256 × 176, voxel size: 0.9 × 0.9 × 0.9 mm ^3^). In all sessions, the first four volumes were discarded to account for magnetic saturation effects. Participants were lying down in the scanner in front of a mirror box that allowed them to see the display of stimuli projected on a screen by an LCD projector. They responded by using a custom-made amagnetic keypad with their right hand. Head movements were minimized by using a vacuum cushion. In each event-related oddball session (I, II, and III), 240 functional volumes were obtained. Before the first oddball fMRI session, the acoustic level of each of the two tones was individually adjusted for optimal comfort during a sham fMRI acquisition. In block-design Session IV, participants were scanned during short periods (±30 s) of navigation in the virtual maze or of SRT task practice, alternating with rest periods (±5–15 s; see Supporting Information); 800 or 720 functional volumes were obtained, respectively.

### fMRI data analysis

Data were pre-processed and analyzed using Statistical Parametric Mapping software SPM2 ( http://www.fil.ion.ucl.ac.uk/spm/software/spm2/; Wellcome Department of Imaging Neuroscience, London, United Kingdom) implemented in MATLAB 6.1 (The MathWorks, Natick, Massachusetts, United States). Pre-processing steps included realignment and adjustment for movement related effects, co-registration of functional and anatomical data, spatial normalization into standard stereotactic MNI space, and spatial smoothing using a Gaussian kernel of 6-mm full width at half maximum (FWHM).

Data were analyzed using a mixed-effects model, aiming at showing a stereotypical effect in the population from which the participants were drawn [ 60]. For each participant, a first-level intra-individual analysis aimed at modeling data to partition observed neurophysiological responses into components of interest, confounds, and error, using a general linear model [ 61]. The effects of interest were then tested by linear contrasts, generating statistical parametric maps [SPM(T)]. Summary statistic images were thresholded at *p* < 0.95 (uncorrected) then further spatially smoothed (6-mm FWHM Gaussian kernel). The second-level analysis consisted of a conjunction analysis, achieved by taking forward a contrast image of the effect of practice in the learning task (Session IV) and a contrast image of the Session (II versus I, or III versus II) or of the Session by Context (spatial versus procedural) effect on brain activity elicited by the presentation of deviant events during the oddball task. Restricted maximum likelihood estimates of variance components were used to allow possible departure from the sphericity assumptions in RFX conjunction analyses [ 62].

In order to test whether offline modifications of neuronal activity were related to the maintenance of the recently acquired memories, as assessed behaviorally, coupling between behavioral performance and BOLD response to deviant events in Session II (versus I) or Session III (versus II) was estimated using a correlation analysis between individual performance levels (or differences in performance levels between learning and retest sessions) and individual statistical parametric maps of effect size at every voxel [ 1, 2]. The statistical parametric maps resulted from main effects contrasts estimating differences in regional BOLD activity from one session to the other (e.g. Session II versus I), computed separately within each learning context.

Furthermore, psychophysiological interaction (PPI) analyses [ 63, 64] were computed in order to test the hypothesis that those areas showing persistent neural activity during oddball sessions after practice of the learned task might gradually (across sessions) establish or reinforce functional connections with other brain regions involved in learning. Coordinates of voxels of interest were determined based on results from RFX analyses described above.

In all the analyses presented above, the resulting set of voxel values for each contrast constituted a map of the t statistic [SPM(T)], thresholded at *p* < 0.001 (uncorrected for multiple comparisons). Statistical inferences were then obtained after corrections at the voxel level using Gaussian random field theory [ 65], either *p* ^corr^ < 0.05 corrected for multiple comparisons in the whole brain volume, or *p* ^svc^ < 0.05, corrected in a small spherical volume (radius 10 mm) around a priori locations of activation in structures of interest, taken from the literature (a list of a priori coordinates is available in Supporting Information).

## Supporting Information

Figure S1 Spatial Learning (A) Mean distance left to destination at the end of the Learning phase (black) and during the Retest phase (red) in the virtual town for each of the 15 participants (S1–S15). Average population values are illustrated on the right side of the panel.
(B) Population's mean performance scores for the five consecutive tests (in their presentation order) during Learning and Retest sessions.
(C) Populations' mean performance scores arranged in such a way that test and retest performances are adjacent for each of the five tests. Note subjective differences in test difficulty, and that improvement over the 1-h interval between learning and test sessions is present for some but not all tests, probably in relation to their varying difficulty.
(10 MB TIF)

Figure S2 Procedural Learning (A) Mean RTs (±SD) for the 30 blocks of the Learning phase (L1–L30) and the nine blocks of the Retest phase (T1–T9). A different, untrained, sequence was presented during blocks L28, T2, and T8, leading to significant performance decrements (i.e. increased RTs; see text).
(B) Mean RTs (±SD) for the first 20 blocks of practice with the learned (blue) and the untrained (red) sequences during fMRI Session IV. RTs for the novel sequence are reliably slower than for the learned sequence, F(1,10) = 40,17, *p* < 0.0001.
(10 MB TIF)

Figure S3 Session-Specific Parameter Estimates (A) Mean parameter estimates (±SEM) of brain responses to deviant auditory events in oddball sessions before (I), immediately after (II), and 45 min after (III) spatial learning, and during delayed virtual navigation (Session IV), in the hippocampus (values averaged across participants over the local maxima within 3 mm from the target location [26 −24 −8 mm]).
(B and C) Mean parameter estimates (±SEM) of brain response (BOLD) to deviant auditory events in oddball sessions before (I), immediately after (II), and 45 min after (III) procedural learning, and during delayed practice of the SRT task (Session IV), in the cerebellum (B) and the caudate nucleus (C) (values averaged across participants over the local maxima within 3 mm from the target location [2 −60 −28 mm] and [−16 0 16] mm, respectively).
(7 MB TIF)

Protocol S1 Supporting Information Text (53 KB DOC)

Table S1 Post-Spatial Training Activity (Main Effects) (153 KB DOC)

Table S2 Post-Procedural Training Activity (Main Effects) (184 KB DOC)

## Abbreviations

BOLD: blood oxygen level-dependent
fMRI: functional magnetic resonance imaging
MRI: magnetic resonance imaging
RT: reaction time
SMA: supplementary motor area
SRT: serial reaction time
