# Supplementary material for: Offline Persistence of Memory-Related Cerebral Activity during Active Wakefulness
Source: PLoS Biol. 2006 Mar 28;4(4):e100. doi: 10.1371/journal.pbio.0040100 (PMC1413571; doi:10.1371/journal.pbio.0040100)
Supplement: Supplementary file 1 [file pbio.0040100.sd001.doc]

Offline Persistence of Memory-Related Cerebral Activity during Active Wakefulness

Philippe Peigneux*£, Pierre Orban*, Evelyne Balteau*, Christian Degueldre*, André Luxen*, Steven Laureys*‡, & Pierre Maquet*‡

* Cyclotron Research Center, University of Liège, Liège, Belgium

‡ Neurology Department, CHU, University of Liège, Liège, Belgium

*£* Email: Philippe.Peigneux@ulg.ac.be	* Website: http://www.ulg.ac.be/crc

SUPPORTING INFORMATION

**Circadian and Sleep Parameters**

Qualitative sleep parameters and circadian chronotype averaged across the previous month were respectively assessed using the Pittsburgh Sleep Quality Index questionnaire [1] and a self-assessment questionnaire to determine morningness-eveningness in human circadian rhythms [2]. Average sleep duration for the month preceding the experiment was 7.3 hours (range 6-10 hours) and median subjective sleep quality was 3 on a 4-step scale (from 1 “poor” to 4 “good”). The median score on the morningness-eveningness circadian rhythms questionnaire was 44 (range 27-56), meaning no subject presented extreme evening or morning chronotype.

The St. Mary's Hospital sleep questionnaire [3] subjectively assessed sleep quality and quantity during the night preceding each half-day of testing. Median sleep duration (7.25 versus 6.85 hours), sleep quality (4.73 versus 4.40 on a 5-step scale from very poor [1] to very good [5]) and number of awakenings per night (0.4 versus 0.6) were similar the night preceding the first versus the second experimental half-day (ps > .35; Wilcoxon tests). Likewise, median sleep duration (7.5 versus 7.00 hours), sleep quality (4.73 versus 4.40) and number of awakenings per night (0.47 versus 0.53) did not differ between the nights preceding the administration of the procedural versus the spatial learning task in the protocol (ps > .11; Wilcoxon test).

Learning Tasks

*Virtual Maze (spatial) Learning Task*

All subjects were trained on one of the two half-days in a virtual town inspired by Maguire et al. [4,5] and already used in Peigneux et al. [6] in a more complex form.

The virtual environment was created and presented using a commercially available computer game (Duke Nukem 3D, 3D Realms Entertainment, Apogee Software Ltd., Garland, TX) on an 800-MHz Pentium-III PC (screen size 17”). Subjects had a color 3D, first-person, view from inside the environment in which they navigated at constant speed using arrow keys to control their moves. The town was composed of two disjoint walking areas (A and B), each divided into three districts, into which urban or rural streets with distinctive walls and objects were incorporated. In each district, one target object was identified by a rotating medallion (e.g. the Buddha statue, see Figure 1 in main text).

The Learning condition took place outside of the scanner, immediately after fMRI Session I and before Session II (see Figure 1 in main text). There were three blocks of exploration, each followed by a block of tests. In the exploration condition, subjects moved freely in walking area A. They were explicitly instructed to learn the spatial layout of streets, districts and object locations, and informed that they would be asked later on to find their way in the virtual town. In the place finding test condition, subjects were designated a starting location in walking area A and were instructed to reach a remote object located in another district of the same walking area, in no more than 90 seconds. After this time had elapsed, the distance remaining between the subject’s actual location and his/her final destination was computed using the shortest possible path (arbitrary units) and used as a quantitative estimate of topographical knowledge (i.e. the shorter the remaining distance to destination, the better the performance). Three exploration sessions were conducted, each for a duration of 7.5 minutes. After the first two exploration sessions, two tests of place finding were administered in order to familiarize the subject with the difficulty of the task. At the end of the third exploration session, five tests were administered and used to compute a measure of performance at the end of practice. Mean distance left to destination was 27.1 (arbitrary units; standard deviation [SD] = 12.5).

The Retest condition also took place outside of the scanner, immediately after fMRI Session III (see Figure 1 in main text), ± one hour after the end of the Learning phase. The five tests administered at the end of the third exploration session in the Learning phase (see above) were administered in a different sequence order. Mean distance left to destination was 21.7 distance units (SD = 12.3). A paired comparison (Student t-test) yielded a slight but significant improvement in performance from Learning to Retest (27.1 versus 21.7 units), t(1,14) = 2.10, p = .05 (Supplementary Figure S1). The number of tests was limited to five within each session in order to minimize further learning during test trials. Another reason for doing this was that the possible number of minimally overlapping paths was limited by the size of the virtual environment. An analysis of variance (ANOVA) conducted on performance scores, with the five consecutive tests (in their presentation order within each session) and the session (Learning versus Retest session) as repeated measure factors, revealed variations in performance from one test to the next (main effect of test repetition F(4, 56)=11.97, p < .001). However this does not provide clear evidence for a linear trend toward significant improvement within session (see Panel B of Supplementary Figure S1). Panel C of Supplementary Figure S1 also shows that within-session variations from one test to another are explained by subjective differences in test difficulty, and that improvement over the one-hour interval between learning and test sessions is present for some but not all tests, probably in relation to their varying difficulty. It is possible that the five tests performed at the end of the learning session provided subjects with feedback that contributed to performance after the one-hour interval for the same tests and that this may explain the slight (though statistically significant) improvement between sessions. Given this possibility, we interpret these results as indicating spatial memory maintenance in the navigation task over a one-hour interval. Individual changes in performance from Learning to Retest were computed as the distance left to destination during the Learning phase minus the distance left to destination during the Test phase (i.e. the higher the value, the more the performance improved). This performance measure was further used in correlation analyses with the off-line Blood Oxygen Level Dependent (BOLD) response during oddball II and III sessions (see below and main text).

Finally, during fMRI Session IV (see Figure 1 in main text), subjects were scanned during navigation practice in the virtual maze. They were lying down in the scanner in front of a mirror box that allowed them to see the display of the virtual environment projected on a screen by an LCD projector, and they navigated using a custom-made amagnetic keypad with their right hand. The block-design session included 50 blocks of exploration within the virtual environment, lasting randomly between 15 and 25 seconds, alternating with rest periods, lasting randomly between 8 and 15 seconds. Half of the blocks of exploration used the familiar virtual town (A), in which subjects were trained and tested in Learning and Retest phases. The other half of the blocks contained a novel, unlearned virtual town (B). Blocks were presented in a similar, pre-defined order to all subjects so that they could not explore the same town (i.e. familiar versus novel) more than two times consecutively. At each block, exploration started from a new location out of five possible starting points in each town. Participants were instructed to explore freely both virtual towns and to learn their spatial layout for further testing (that was not administered), in the same way as during behavioral conditions.

*Serial Reaction Time (procedural) Learning Task*

All subjects were trained on the other half-day to the serial reaction time (SRT) task, adapted from Destrebecqz et al. [7,8] and already used by our group [9-11] in its probabilistic [12] form.

Before the SRT task, participants were simply told that the aim of the experiment was to study the cerebral effects of sustained practice on a simple motor task. Participants faced a screen where four permanent position markers were displayed horizontally above four spatially compatible response keys. A single SRT block consisted of 96 successive trials. On each trial, a black dot appeared 2 cm below one of the position markers, and the task consisted of pressing as fast and as accurately as possible with the right hand on the corresponding key. The next stimulus was then displayed after a 0 ms response-stimulus interval (RSI). Such a null (0 ms) RSI was chosen because it makes sequential learning more implicit in nature [8]. Errors were indicated by a visual display. Not indicated to participants, each block contained 8 repetitions of one of the two following 12-element sequences: 342312143241 (S1) or 341243142132 (S2). These sequences were identical insofar as stimulus locations and transitions frequency were concerned, but differed in terms of which sub-sequences of three elements they contained. Each block was followed by a short break, during which mean reaction time and accuracy level were displayed on the screen.

The Learning condition took place outside of the scanner, immediately after fMRI Session I and before Session II (see Figure 1 in main text). In this condition, 30 blocks of SRT practice (L1-L30) were administered using one of the two sequences. A one-way analysis of variance (ANOVA) indicated that mean (± SD) reaction times gradually decreased with practice from block 1 (553 ± 81.4 ms) to block 30 (286 ± 58 ms), F(29,406) = 57.06; p<0.0001, indicating visuo-motor improvement on the task (Supplementary Figure S2, Panel A). In order to assess the extent to which subjects additionally learned the regularities of the trained sequence, the other sequence was presented during block L28. Assuming that reaction time improvement reflects motor response preparation and anticipation of the next stimulus, reaction times should have increased on block L28 only if participants had acquired specific knowledge about the sequential regularities characteristic of the trained sequence presented over blocks L1-L27 [13]. Indeed, a planned comparison yielded a significant increase in reaction time (average 209 ms) from the learned sequence on block L27 (309 ± 49 ms) to the unlearned sequence on block L28 (518 ± 58 ms), F(1,15) = 140.55; p<0.001 (Supplementary Figure S2, Panel A).

The Retest condition also took place outside of the scanner, immediately after fMRI Session III (see Figure 1 in main text). Nine blocks of SRT practice (T1-T9) were administered using the sequence trained in the Learning phase, except blocks T2 and T8, which used the other sequence. A two-way analysis of variance (ANOVA) using blocks L27-L28 and T1-T2 with Phase [Learning versus Retest] and Sequence type [Trained versus Untrained] factors disclosed a significant effect of Phase, F(1, 14)=35,291, p < .0001, a significant effect of Sequence type, F(1, 14)=173,39, p < .00001, and a significant [Phase X Sequence type] interaction F(1, 14)=12,746, p < .005. Post-hoc analyses (HSD Tukey) indicated that reaction times improved from the Learning to the Retest phase for the untrained (518 versus 458 ms, p < .005) sequence, but not for the learned sequence (309 versus 298 ms, p > .5). The difference between the trained and untrained sequence was significant within both Learning and Retest sessions (ps < .0005; Supplementary Figure S2, Panel A). These results suggest that only the visuo-motor component of learning improved between sessions after a one-hour interval. This was at variance with the procedural knowledge of the sequential regularities, which remained stable between sessions. Individual levels of sequence knowledge during Learning and Retest sessions were separately estimated based on the differences between reaction times for the trained and the untrained sequences (L28 minus L27 and T2 minus T1, respectively; i.e. higher values mean better sequence knowledge). These measures were further used in correlation analyses with the offline Blood Oxygen Level Dependent (BOLD) response during oddball sessions II and III (see main text).

Finally, in fMRI Session IV (see Figure 1 in main text), subjects were scanned during practice of the SRT task. Subjects were lying down in the scanner in front of a mirror box that allowed them to see the display of stimuli projected on a screen by an LCD projector. Subjects responded by using a custom-made amagnetic keypad with their right hand. The block-design session included 60 blocks of SRT practice, lasting randomly from 2 to 3 sequences (exact duration was determined by the subject’s response rapidity, given the use of fixed 0 ms response-stimulus interval), alternating with rest periods, lasting randomly from 5 to 15 seconds, during which mean reaction time and accuracy level were displayed on screen. One third of the SRT blocks used the learned sequence, while the other two thirds used the other, untrained, sequence. No more than three consecutive blocks contained the same sequence. Panel B of Supplementary Figure S2 illustrates the evolution of RTs across the first 20 blocks of the learned and the novel sequence during fMRI Session IV. It shows that RTs are reliably faster for the learned than the unlearned sequence, F(1,10)=40,17, p < .0001, a difference that remained stable all through the session (Sequence by Block repetition interaction effect F(19,190) = .91, p = .58). RTs at the beginning of fMRI session IV (452 ms) were slower than RTs observed at the end of Retest (321 ms), then they become progressively faster with practice, F(19,190) = 4.12, p < .0001, the initial slowing being due to the necessity for the subjects to adapt to the new requirements of the SRT task in the fMRI scanner (different keyboard, lying down position, environmental noise …).

fMRI Data Analysis

Data were pre-processed and analyzed using Statistical Parametric Mapping software SPM2 (http://www.fil.ion.ucl.ac.uk/spm/software/spm2/; Wellcome Department of Imaging Neuroscience, London, UK) implemented in MATLAB 6.1 (Mathworks Inc., Sherbom, MA). Pre-processing included realignment and adjustment for movement related effects, co-registration of functional and anatomical data, spatial normalization into standard stereotactic MNI space, and spatial smoothing using a Gaussian kernel of 6 mm full width at half maximum (FWHM).

Mixed-effects analyses

Data were analyzed using a mixed-effects model, aiming at showing a stereotypical effect in the population from which the subjects were drawn [14]. This procedure was implemented in two processing steps accounting for fixed and random effects, respectively.

For each subject, a first-level intra-individual analysis aimed at modeling data to partition observed neurophysiological responses into components of interest, confounds and error, using a general linear model [15]. All sessions were modeled together. The regressors of interest were built using stick functions (oddball sessions) or box cars (procedural or spatial task practice sessions), positioned at each stimulus or block presentation. These regressors were secondarily convolved with the canonical hemodynamic response function. Movement parameters derived from realignment of the functional volumes (translations in x, y and z directions and rotations around x, y and z axes) were included as covariates of no interest in the design matrix. High-pass filtering was implemented in the matrix design using a cut-off period of 128 seconds to remove low drift frequencies from the time series. Serial correlations were estimated with a restricted maximum likelihood (ReML) algorithm using an intrinsic autoregressive model during parameter estimation. The effects of interest were then tested by linear contrasts, generating statistical parametric maps [SPM(T)]. Since no inference was made at this (fixed effect) level of analysis, summary statistic images were thresholded at p < 0.95 (uncorrected).

Within each oddball session (I, II, III) in the context of specific task practice (spatial or procedural), an event-related analysis estimated Blood Oxygen Level Dependent (BOLD) responses evoked by the auditory presentation of deviant tones. Simple linear contrasts looked for the main effect of auditory deviant events within each session. Session effects were assessed, looking for the differential main effect of deviant events between sessions (II versus I, or III versus II, or III versus I) within each task context (i.e. half-day with spatial or procedural learning). Finally, Session [{II versus I} or {III versus II} or {III versus I}] by Context [spatial versus procedural] interaction contrasts looked for the differential main effect of deviant events between sessions modulated by the context of the learning task experienced that specific half-day. Within fMRI Session IV, acquired during practice of spatial or procedural learning tasks, BOLD responses were estimated using a boxcar function. Linear contrasts estimated respectively the main effect of exploration in the town or of SRT practice.

Individual summary statistic images obtained at the first level were further spatially smoothed (6 mm FWHM Gaussian kernel). The second-level analysis consisted of a conjunction analysis, achieved by taking forward a contrast image of the effect of practice in the learning task (session IV) and a contrast image of the Session (II versus I, or III versus II) or of the Session by Context [spatial versus procedural] effect. This analysis aimed at assessing the brain areas in which activity was both (a) modified at a given time point during oddball practice (II or III) as a function of prior experience of a particular learning task (spatial or procedural) and (b) present during practice of this particular learning task. Restricted maximum likelihood estimates of variance components were used to allow possible departure from the sphericity assumptions in RFX conjunction analyses [16].

The resulting set of voxel values for each contrast constituted a map of the t statistic [SPM(T)], thresholded at p<0.001 (uncorrected for multiple comparisons). Statistical inferences were then obtained after corrections at the voxel level using Gaussian random field theory [17], either p^corr^ < .05 corrected for multiple comparisons in the whole brain volume or p^svc(10mm)^ < .05 corrected in a small spherical volume around a priori locations of activation in structures of interest, taken from the literature (see list below) .

Psychophysiological interaction analyses

Psychophysiological interaction (PPI) analyses [18,19] were computed to test the hypothesis that those areas showing persistent neural activity during oddball sessions after practice of the learned task might gradually (across sessions) establish or reinforce functional connections with other brain regions involved in learning. The computational procedure was as follows, adapted from [20]. Coordinates of voxels of interest were determined based on results from RFX analyses described above.

For each subject, we computed at the individual level the oddball session [{II versus I} or {III versus II} or {III versus I}] contrast effect (corresponding to the summary statistic images entered in the RFX analysis) and determined the local maximum of activation in a small spherical volume around voxels of interest (radius 6 mm). This peak value was selected, unless identified outside of the brain structure of interest upon visual inspection of the individual normalized anatomical T1 image, in which case the maximum value that fitted the anatomical location was selected. Individual time series for each of these locations were obtained separately for each oddball session by extracting the first principal component [21] from all raw voxel time series in a sphere (radius 4 mm) centered on the coordinate of the subject-specific peak value. These time series were mean-corrected and high-pass filtered to remove low-frequency signal drifts. Then, the hemodynamic response function was deconvolved from the BOLD time series to keep the underlying neuronal time series [18].

Next, PPI analyses were prepared separately for each location of interest at the individual level. Intra-individual analyses were computed using the same constraints as specified above (i.e. 1st level in main RFX analysis). In addition to the neuronal time series from each oddball session (I, II, III) within a specific learning task context (spatial or procedural), the model of connectivity included movement parameters as effects of no interest. Next, individual summary statistic images obtained at the first level (fixed effects) analysis were spatially smoothed (6mm FWHM Gaussian kernel) and entered into a second-level (random effects) analysis using one sample t-tests. The resulting set of voxel values was thresholded at p<0.001 (uncorrected). Statistical inferences were obtained using Gaussian random field theory [17] after corrections at the voxel level in a small spherical volume (radius 10 mm) around a priori locations of activation in structures of interest, taken from the literature (see list below) .

A priori locations of interest and activations localization

Localization of activation peaks was performed based on stereotactic MRI atlases of the human brain [22,23]. Nomenclature for cerebellum locations is based on [24]. Standard stereotactic coordinates of previously published a priori locations, used for small volume corrections, are as follows:

[A] Locations involved in actual and offline spatial/topographical learning: right hippocampus: 30 –16 –22, 30 –20 –16 mm [4] and 28 –28 –16, 28 –28 –12 mm [6]; left hippocampus: –16 –26 –6 [4]; right parahippocampal gyrus: 22 –40 –8, 22 –30 –20 [25], 24 –33 –18, 30 –45 –12 [26], 20 –46 –12 [27], 21 –42 –6 mm [28]; left parahippocampal gyrus: –21 –42 –15 [26], –22 –38 –12 [27], –18 –42 –9 mm [28]; superior frontal gyrus –10 66 8 mm [4]; retrosplenial cortex 14 –56 8 [29,30]

[B] Locations involved in actual and offline motor procedural learning: medial cerebellum –1 –57 –20 mm [31] and lateral cerebellum 32 –66 –30 mm [32]; supplementary motor area (SMA) 9 –2 57 mm [33]; caudate nucleus of the striatum –16 8 10 mm [11] and –16 –6 24 mm [10]; putamen 22 0 0 mm [33]; dorsal premotor cortex –38 4 46 mm [33].

**Supporting Information References**

1. Buysse DJ, Reynolds CF, 3rd, Monk TH, Berman SR, Kupfer DJ (1989) The Pittsburgh Sleep Quality Index: a new instrument for psychiatric practice and research. Psychiatry Res 28: 193-213.

2. Horne JA, Ostberg O (1976) A self-assessment questionnaire to determine morningness-eveningness in human circadian rhythms. Int J Chronobiol 4: 97-110.

3. Ellis BW, Johns MW, Lancaster R, Raptopoulos P, Angelopoulos N, et al. (1981) The St. Mary's Hospital sleep questionnaire: a study of reliability. Sleep 4: 93-97.

4. Maguire EA, Burgess N, Donnett JG, Frackowiak RS, Frith CD, et al. (1998) Knowing where and getting there: A human navigation network. Science 280: 921-924.

5. Burgess N, Maguire E, O'Keefe J (2002) The human hippocampus and spatial and episodic memory. Neuron 35: 625.

6. Peigneux P, Laureys S, Fuchs S, Collette F, Perrin F, et al. (2004) Are spatial memories strengthened in the human hippocampus during slow wave sleep? Neuron 44: 535-545.

7. Destrebecqz A, Peigneux P, Laureys S, Degueldre C, Del Fiore G, et al. (2005) The neural correlates of implicit and explicit sequence learning: Interacting networks revealed by the process dissociation procedure. Learn Mem 12: 480-490.

8. Destrebecqz A, Cleeremans A (2001) Can sequence learning be implicit? New evidence with the process dissociation procedure. Psychon Bull Rev 8: 343-350.

9. Maquet P, Laureys S, Peigneux P, Fuchs S, Petiau C, et al. (2000) Experience-dependent changes in cerebral activation during human REM sleep. Nature Neurosci 3: 831-836.

10. Peigneux P, Laureys S, Fuchs S, Destrebecqz A, Collette F, et al. (2003) Learned material content and acquisition level modulate cerebral reactivation during posttraining rapid-eye-movements sleep. NeuroImage 20: 125-134.

11. Peigneux P, Maquet P, Meulemans T, Destrebecqz A, Laureys S, et al. (2000) Striatum forever despite sequence learning variability: a random effect analysis of PET data. Hum Brain Mapp 10: 179-194.

12. Jimenez L, Mendez C, Cleeremans A (1996) Comparing direct and indirect measures of sequence learning. J Exp Psychol Learn Mem Cogn 22: 948-969.

13. Reed J, Johnson P (1994) Assessing implicit learning with indirect tests: Determining what is learned about sequence structure. J Exp Psychol Learn Mem Cogn 20: 585-594.

14. Penny W, Holmes A (2003) Random-effect analysis. In: Frackowiak R, Friston K, Frith C, Dolan R, Price C et al., editors. Human brain function. 2nd ed. London: Academic Press.

15. Friston K (2003) Introduction: experimental design and statistical parametric mapping. In: Frackowiak R, Friston K, Frith C, Dolan R, Price C et al., editors. Human brain function. 2nd ed. London: Academic Press.

16. Friston KJ, Glaser DE, Henson RN, Kiebel S, Phillips C, et al. (2002) Classical and Bayesian inference in neuroimaging: applications. Neuroimage 16: 484-512.

17. Worsley KJ (1996) A unified statistical approach for determining significant signals in images of cerebral activation. Hum Brain Mapp 4: 58-73.

18. Gitelman DR, Penny WD, Ashburner J, Friston KJ (2003) Modeling regional and psychophysiologic interactions in fMRI: the importance of hemodynamic deconvolution. Neuroimage 19: 200-207.

19. Friston KJ, Buechel C, Fink GR, Morris J, Rolls E, et al. (1997) Psychophysiological and modulatory interactions in neuroimaging. Neuroimage 6: 218-229.

20. Stephan KE, Marshall JC, Friston KJ, Rowe JB, Ritzl A, et al. (2003) Lateralized cognitive processes and lateralized task control in the human brain. Science 301: 384-386.

21. Buchel C, Friston KJ (1997) Modulation of connectivity in visual pathways by attention: Cortical interactions evaluated with structural equation modelling and fMRI. Cereb Cortex 7: 768-778.

22. Duvernoy HM (1999) The human brain: Surface, Three-dimensional sectional anatomy. With MRI, and blood supply. New-York: Springer-Verlag. 491 p.

23. Talairach J, Tournoux P (1988) Co-Planar Stereotaxic Atlas of the Human Brain. New York: Thieme. 122 p

24. Schmahmann JD, Doyon J, Toga A, Petrides M, Evans A (2000) MRI atlas of the human cerebellum. San Diego, CA: Academic Press. 167 p.

25. Maguire EA, Frith CD, Burgess N, Donnett JG, OKeefe J (1998) Knowing where things are: Parahippocampal involvement in encoding object locations in virtual large-scale space. J Cogn Neurosci 10: 61-76.

26. Burgess N, Maguire EA, Spiers HJ, O'Keefe J (2001) A temporoparietal and prefrontal network for retrieving the spatial context of lifelike events. Neuroimage 14: 439-453.

27. Maguire EA, Frith CD, Cipolotti L (2001) Distinct neural systems for the encoding and recognition of topography and faces. Neuroimage 13: 743-750.

28. Shelton AL, Gabrieli JD (2002) Neural correlates of encoding space from route and survey perspectives. J Neurosci 22: 2711-2717.

29. Maguire EA (2001) The retrosplenial contribution to human navigation: a review of lesion and neuroimaging findings. Scand J Psychol 42: 225-238.

30. Ghaem O, Mellet E, Crivello F, Tzourio N, Mazoyer B, et al. (1997) Mental navigation along memorized routes activates the hippocampus, precuneus, and insula. Neuroreport 8: 739-744.

31. Lafleur MF, Jackson PL, Malouin F, Richards CL, Evans AC, et al. (2002) Motor Learning Produces Parallel Dynamic Functional Changes during the Execution and Imagination of Sequential Foot Movements. Neuroimage 16: 142-157.

32. Penhune VB, Doyon J (2002) Dynamic cortical and subcortical networks in learning and delayed recall of timed motor sequences. J Neurosci 22: 1397-1406.

33. Doyon J, Penhune V, Ungerleider LG (2003) Distinct contribution of the cortico-striatal and cortico-cerebellar systems to motor skill learning. Neuropsychologia 41: 252-262.
