# Supplementary material for: Offline Persistence of Memory-Related Cerebral Activity during Active Wakefulness
Source: PLoS Biol. 2006 Mar 28;4(4):e100. doi: 10.1371/journal.pbio.0040100 (PMC1413571; doi:10.1371/journal.pbio.0040100)
Supplement: Supplementary file 2 [file pbio.0040100.st001.doc]

SUPPLEMENTARY TABLE S1. POST-SPATIAL TRAINING ACTIVITY (1/4)

|  | | INCREASED ACTIVITY | | | | | | | | | | DECREASED ACTIVITY | | | | | | | | |
| --- | --- | --- | --- | --- | --- | --- | --- | --- | --- | --- | --- | --- | --- | --- | --- | --- | --- | --- | --- | --- |
|  | | IMMEDIATE (II > I) | | | | | DELAYED (III > II) | | | | | IMMEDIATE (I > II) | | | | | DELAYED (II > III) | | | |
|  | *x* | | *y* | *z* | *Z* | *x* | | *y* | *z* | *Z* | *x* | | *y* | *z* | *Z* | *x* | | *y* | *z* | *Z* |
| Hippocampus | 28 | | -22 | -8 | 5.29 a |  | |  |  |  |  | |  |  |  | 24 | | -22 | -14 | 3.69 ag |
|  | -22 | | -24 | -2 | 3.50 a |  | |  |  |  |  | |  |  |  | -22 | | -22 | -12 | 3.47 a |
| Parahippocampal Gyrus | 14 | | -36 | -6 | 3.61 a |  | |  |  |  |  | |  |  |  |  | |  |  |  |
|  | -16 | | -34 | -6 | 3.16 a | -26 | | -34 | -12 | 3.54 b |  | |  |  |  | -8 | | -44 | -2 | 3.72 g |
|  | 28 | | -28 | -20 | 3.38 a* | -34 | | -38 | -14 | 3.11 |  | |  |  |  |  | |  |  |  |
| Retrosplenial Cortex | 6 | | -48 | 12 | 3.67 a |  | |  |  |  |  | |  |  |  | 6 | | -44 | 12 | 3.36 a |
| Inferior Occipital Gyrus | -36 | | -90 | -14 | 3.92 |  | |  |  |  |  | |  |  |  | 46 | | -72 | -6 | 3.69 g |
| Middle Occipital Gyrus | -44 | | -80 | -14 | 3.80 a |  | |  |  |  | 46 | | -84 | 8 | 3.34 | -48 | | -78 | -12 | 3.84 ag |
|  |  | |  |  |  |  | |  |  |  |  | |  |  |  | 48 | | -62 | -8 | 3.15 |
| Superior Occipital Gyrus |  | |  |  |  |  | |  |  |  |  | |  |  |  |  | |  |  |  |
| Fusiform Gyrus | 44 | | -42 | -22 | 3.25 |  | |  |  |  |  | |  |  |  | 24 | | -42 | -22 | 3.37 |
|  |  | |  |  |  |  | |  |  |  |  | |  |  |  | -46 | | -60 | -24 | 3.42 |
| Lingual Gyrus |  | |  |  |  |  | |  |  |  | 14 | | -88 | -24 | 4.35 |  | |  |  |  |
| Cuneus | -24 | | -82 | 4 | 3.71 a |  | |  |  |  |  | |  |  |  | -20 | | -86 | 28 | 3.45 f |
| Inferior Temporal Gyrus |  | |  |  |  |  | |  |  |  |  | |  |  |  |  | |  |  |  |
| Middle Temporal Gyrus | -44 | | -80 | -14 | 3.80 a |  | |  |  |  | 52 | | -72 | -8 | 3.91 | -48 | | -78 | -12 | 3.84 a |
|  |  | |  |  |  |  | |  |  |  |  | |  |  |  | 40 | | -74 | 18 | 3.55dfg |
|  |  | |  |  |  |  | |  |  |  |  | |  |  |  | -52 | | -78 | 12 | 3.48 df |

SUPPLEMENTARY TABLE S1. POST-SPATIAL TRAINING ACTIVITY – CONTINUED (2/4)

|  |  |  |  |  |  |  |  |  |  |  |  |  | 44 | -74 | -8 | 3.18 a |
| --- | --- | --- | --- | --- | --- | --- | --- | --- | --- | --- | --- | --- | --- | --- | --- | --- |
|  |  |  |  |  |  |  |  |  |  |  |  |  | -52 | -76 | 12 | 3.28 fg |
| Superior Temporal Gyrus |  |  |  |  |  |  |  |  |  |  |  |  |  |  |  |  |
| Cingulate Gyrus | 2 | 14 | 44 | 4.19 a |  |  |  |  | 20 | -34 | 44 | 3.55 | 2 | -54 | 2 | 3.91 |
|  | 12 | -40 | 42 | 3.76 | 12 | -28 | 40 | 3.71 |  |  |  |  | 18 | -14 | 42 | 3.43 |
|  | 28 | -68 | 4 | 3.76 |  |  |  |  |  |  |  |  |  |  |  |  |
|  | -12 | -18 | 36 | 5.14 |  |  |  |  |  |  |  |  |  |  |  |  |
| Inferior Parietal Lobule | -34 | -38 | 44 | 4.63 |  |  |  |  |  |  |  |  |  |  |  |  |
|  | 38 | -54 | 54 | 3.47 |  |  |  |  |  |  |  |  |  |  |  |  |
| Supramarginal Gyrus |  |  |  |  |  |  |  |  |  |  |  |  |  |  |  |  |
| *Angular Gyrus* |  |  |  |  | 48 | -76 | 32 | 3.17 |  |  |  |  |  |  |  |  |
| Superior Parietal Lobule | -38 | -54 | 58 | 3.82 a |  |  |  |  |  |  |  |  | -18 | -62 | 68 | 3.22 |
| Paracentral Lobule |  |  |  |  |  |  |  |  |  |  |  |  | -26 | -36 | 46 | 3.41 |
| Postcentral Gyrus | 4 | -46 | 68 | 3.41 a |  |  |  |  |  |  |  |  | -10 | -46 | 76 | 3.57 |
|  | -54 | -12 | 50 | 5.15 |  |  |  |  |  |  |  |  | -30 | -32 | 46 | 3.86 df |
| Precentral Gyrus | -54 | 0 | 34 | 5.70 |  |  |  |  |  |  |  |  |  |  |  |  |
| Precuneus | 12 | -72 | 38 | 3.48 | 32 | -72 | 38 | 3.30 |  |  |  |  |  |  |  |  |
|  | -10 | -62 | 56 | 3.27 | -34 | -72 | 38 | 4.11 | -32 | -72 | 40 | 4.38 |  |  |  |  |
| Inferior Frontal Gyrus | -60 | 8 | 26 | 4.22 | 50 | 46 | 16 | 3.44 | 56 | 36 | 14 | 3.41 |  |  |  |  |
|  | 62 | 14 | 22 | 5.66 | -44 | 8 | 28 | 3.92 |  |  |  |  |  |  |  |  |
|  |  |  |  |  | 52 | 8 | 20 | 5.00 |  |  |  |  |  |  |  |  |
| Middle Frontal Gyrus | 38 | -4 | 58 | 5.02 adf | -50 | 6 | 42 | 3.87 |  |  |  |  | 30 | -10 | 48 | 3.38 a |
|  | -18 | -2 | 64 | 5.81 |  |  |  |  |  |  |  |  | -42 | -30 | 30 | 3.44 |
|  |  |  |  |  |  |  |  |  |  |  |  |  | 28 | -10 | 48 | 3.69 |

SUPPLEMENTARY TABLE S1. POST-SPATIAL TRAINING ACTIVITY – CONTINUED (3/4)

| Medial Frontal Gyrus |  |  |  |  |  |  |  |  |  |  |  |  | 12 | 34 | 36 | 3.96 |
| --- | --- | --- | --- | --- | --- | --- | --- | --- | --- | --- | --- | --- | --- | --- | --- | --- |
| SMA | 14 | 2 | 68 | 5.85 adf |  |  |  |  |  |  |  |  | 10 | 2 | 56 | 3.27 dg |
|  | -12 | 2 | 56 | 6.04 |  |  |  |  |  |  |  |  | 10 | 4 | 62 | 3.18 af |
| preSMA |  |  |  |  |  |  |  |  |  |  |  |  | 8 | 12 | 62 | 3.79 dfg |
| Superior Frontal Gyrus | 20 | 14 | 62 | 4.94 a |  |  |  |  |  |  |  |  | 14 | 14 | 62 | 3.09 ad |
|  | 8 | 8 | 60 | 6.42 |  |  |  |  |  |  |  |  |  |  |  |  |
|  | -6 | 8 | 56 | 6.10 |  |  |  |  |  |  |  |  |  |  |  |  |
| Insula | -24 | 20 | -4 | 5.39 |  |  |  |  | -30 | 2 | -6 | 3.93 |  |  |  |  |
|  | 30 | 14 | 0 | 5.42 |  |  |  |  |  |  |  |  |  |  |  |  |
|  | -30 | 24 | 0 | 5.61 | -36 | 20 | 16 | 3.94 |  |  |  |  |  |  |  |  |
|  | -44 | -32 | 20 | 4.22 |  |  |  |  |  |  |  |  |  |  |  |  |
|  | 32 | 22 | 14 | 3.79 |  |  |  |  |  |  |  |  |  |  |  |  |
| Thalamus | 12 | -12 | 10 | 5.54 a |  |  |  |  |  |  |  |  |  |  |  |  |
|  | -10 | -12 | 8 | 5.88 f |  |  |  |  |  |  |  |  |  |  |  |  |
| Brainstem | 6 | -20 | -14 | 6.31 |  |  |  |  |  |  |  |  | 8 | -22 | -16 | 4.83 |
|  | -12 | -10 | -2 | 5.72 |  |  |  |  |  |  |  |  | -8 | -30 | 0 | 3.81 |
|  | -2 | -24 | -34 | 4.90 |  |  |  |  |  |  |  |  | -6 | -22 | -16 | 3.63 |
| Lateral Geniculum Body | -22 | -24 | -2 | 3.50 |  |  |  |  |  |  |  |  |  |  |  |  |
| Caudate Nucleus | 14 | 4 | 10 | 4.06 |  |  |  |  |  |  |  |  |  |  |  |  |
| Putamen | -20 | 0 | 8 | 4.81 f | -24 | -2 | 18 | 4.72 f |  |  |  |  |  |  |  |  |
|  | -20 | 8 | 6 | 4.61 | 24 | 16 | 10 | 4.08 |  |  |  |  |  |  |  |  |
| Globus Pallidus |  |  |  |  | 18 | -4 | 0 | 4.38 b |  |  |  |  |  |  |  |  |
|  |  |  |  |  | -16 | -12 | -4 | 3.82 |  |  |  |  |  |  |  |  |
| Cerebellum – fastigial nucleus | |  |  |  |  |  |  |  | 2 | -60 | -28 | 5.42 c |  |  |  |  |

SUPPLEMENTARY TABLE S1. POST-SPATIAL TRAINING ACTIVITY – CONTINUED (4/4)

| Cerebellum – Crus I | -42 | -48 | -34 | 3.99 f |  |  |  |  | 30 | -76 | -28 | 3.48 f | -42 | -46 | -38 | 4.02 d |
| --- | --- | --- | --- | --- | --- | --- | --- | --- | --- | --- | --- | --- | --- | --- | --- | --- |
|  |  |  |  |  |  |  |  |  |  |  |  |  | -44 | -48 | -30 | 3.71 |
| Cerebellum – area VI | -28 | -64 | -30 | 3.88 f |  |  |  |  | -10 | -68 | -12 | 3.96 | -8 | -44 | -2 | 3.72 |
|  | 34 | -38 | -36 | 4.35 a |  |  |  |  |  |  |  |  | 36 | -38 | -36 | 3.63 af |
| Cerebellum – area VIIIA | -12 | -64 | -38 | 4.36 | 10 | -68 | -42 | 3.87 |  |  |  |  | -36 | -50 | -48 | 3.85 |
| Cerebellum – area IX | -16 | -54 | -44 | 3.86 |  |  |  |  |  |  |  |  |  |  |  |  |
|  | 16 | -56 | -42 | 3.22 |  |  |  |  |  |  |  |  | -6 | -54 | -50 | 3.60 |
| Cerebellum – area X |  |  |  |  |  |  |  |  |  |  |  |  | 22 | -38 | -44 | 3.36 |

a : area activated in the [session II versus I] by learning [spatial versus procedural] showing immediate post-spatial learning activity increase (Table 1a)

b : area activated in the [session III versus II] by learning [spatial versus procedural] showing delayed post-spatial learning activity increase (Table 1c)

c : area activated in the [session II versus I] by learning [procedural versus spatial] showing immediate post-procedural learning activity increase (Table 2a)

d : area activated in the [session III versus II] by learning [procedural versus spatial] showing delayed post-procedural learning activity increase (Table 2c)

e : area activated in the [session II versus III] by learning [procedural versus spatial] showing delayed post-procedural learning activity decrease (Table 2d)

f : area activated in the [session I versus II] by learning [procedural versus spatial] showing immediate post-procedural learning activity decrease (Table 2b)

g : area activated in the [session II versus III] by learning [spatial versus procedura] showing delayed post-spatial learning activity decrease (Table 1d)
