# Supplementary material for: Offline Persistence of Memory-Related Cerebral Activity during Active Wakefulness
Source: PLoS Biol. 2006 Mar 28;4(4):e100. doi: 10.1371/journal.pbio.0040100 (PMC1413571; doi:10.1371/journal.pbio.0040100)
Supplement: Supplementary file 3 [file pbio.0040100.st002.doc]

SUPPLEMENTARY TABLE S2. POST-PROCEDURAL TRAINING ACTIVITY (1/4)

|  | INCREASED ACTIVITY | | | | | | | | | | DECREASED ACTIVITY | | | | | | | | | |
| --- | --- | --- | --- | --- | --- | --- | --- | --- | --- | --- | --- | --- | --- | --- | --- | --- | --- | --- | --- | --- |
|  | | IMMEDIATE (II > I) | | | | DELAYED (III > II) | | | | IMMEDIATE (I > II) | | | | | DELAYED (II > III) | | | |  | |
|  | | *x* | *y* | *z* | *Z* | *x* | *y* | *z* | *Z* | *x* | | *y* | *z* | *Z* | *x* | *y* | *z* | *Z* | |  |
| Hippocampus | |  |  |  |  |  |  |  |  |  | |  |  |  |  |  |  |  | |  |
| Parahippocampal Gyrus | |  |  |  |  |  |  |  |  |  | |  |  |  |  |  |  |  | |  |
| Retrosplenial Cortex | |  |  |  |  |  |  |  |  |  | |  |  |  |  |  |  |  | |  |
| Inferior Occipital Gyrus | |  |  |  |  |  |  |  |  | -52 | | -70 | 0 | 4.76 |  |  |  |  | |  |
| Middle Occipital Gyrus | |  |  |  |  | 42 | -80 | 10 | 3.69 | -50 | | -78 | -4 | 4.38 a |  |  |  |  | |  |
|  | |  |  |  |  |  |  |  |  | 54 | | -68 | -12 | 5.09 |  |  |  |  | |  |
|  | |  |  |  |  |  |  |  |  | 44 | | -78 | 12 | 5.04 f |  |  |  |  | |  |
| Superior Occipital Gyrus | |  |  |  |  |  |  |  |  | -32 | | -84 | 30 | 3.41 | -30 | -84 | 26 | 3.37 | |  |
|  | |  |  |  |  |  |  |  |  | 36 | | -80 | 26 | 4.56 |  |  |  |  | |  |
| Fusiform Gyrus | |  |  |  |  |  |  |  |  | -28 | | -74 | -24 | 4.66 f | -24 | -68 | -16 | 3.60 | |  |
| Lingual Gyrus | |  |  |  |  |  |  |  |  | 4 | | -72 | 2 | 3.40 |  |  |  |  | |  |
|  | |  |  |  |  |  |  |  |  | -8 | | -74 | -10 | 3.41 f |  |  |  |  | |  |
| Cuneus | |  |  |  |  | -20 | -78 | 6 | 3.41 | -12 | | -82 | 8 | 3.26 af | 20 | -68 | -14 | 4.18 | |  |
|  | |  |  |  |  |  |  |  |  | -20 | | -82 | 18 | 3.86 f | 10 | -82 | 4 | 3.28 | |  |
| Inferior Temporal Gyrus | |  |  |  |  |  |  |  |  |  | |  |  |  |  |  |  |  | |  |
| Middle Temporal Gyrus | | 56 | -38 | 6 | 5.39 | 54 | -72 | 16 | 3.40 | 50 | | -74 | -16 | 3.69 |  |  |  |  | |  |
|  | |  |  |  |  | 40 | -76 | 10 | 3.42 d | -52 | | -74 | -8 | 3.56 a |  |  |  |  | |  |
|  | |  |  |  |  | -44 | -72 | 14 | 4.42 d | 44 | | -78 | 12 | 5.04 dfg |  |  |  |  | |  |

SUPPLEMENTARY TABLE S2. POST-PROCEDURAL TRAINING ACTIVITY – CONTINUED (2/4)

|  |  |  |  |  |  |  |  |  | -46 | -76 | 16 | 4.35 dfg |  |  |  |  |
| --- | --- | --- | --- | --- | --- | --- | --- | --- | --- | --- | --- | --- | --- | --- | --- | --- |
| Superior Temporal Gyrus | 68 | -34 | 16 | 3.59 |  |  |  |  | 56 | 14 | -2 | 3.51 |  |  |  |  |
|  | -58 | -44 | 12 | 3.88 |  |  |  |  | -58 | -30 | 24 | 3.30 f |  |  |  |  |
| Cingulate Gyrus | -8 | 16 | 48 | 3.19 | -2 | 2 | 46 | 3.35 | -12 | -2 | 48 | 3.38 | -2 | -50 | 2 | 3.60 |
|  |  |  |  |  | 2 | 16 | 42 | 3.31 | 2 | -48 | 2 | 4.34 |  |  |  |  |
|  |  |  |  |  | -18 | -8 | 46 | 5.73 | -46 | -2 | 28 | 3.69 |  |  |  |  |
|  |  |  |  |  |  |  |  |  | 2 | -2 | 50 | 3.22 |  |  |  |  |
|  |  |  |  |  |  |  |  |  | -20 | -8 | 44 | 4.53 |  |  |  |  |
| Inferior Parietal Lobule |  |  |  |  | -46 | -40 | 34 | 3.38 | -36 | -54 | 58 | 3.16 |  |  |  |  |
|  | 44 | -40 | 46 | 3.48 | 50 | -36 | 26 | 3.76 | -64 | -26 | 24 | 3.54 |  |  |  |  |
| Supramarginal Gyrus |  |  |  |  |  |  |  |  |  |  |  |  |  |  |  |  |
| *Angular Gyrus* |  |  |  |  |  |  |  |  |  |  |  |  |  |  |  |  |
| Superior Parietal Lobule |  |  |  |  | -22 | -72 | 44 | 5.08 | -30 | -50 | 62 | 3.45 a |  |  |  |  |
|  |  |  |  |  | 20 | -54 | 64 | 3.21 | 28 | -50 | 62 | 6.15 f |  |  |  |  |
|  |  |  |  |  |  |  |  |  | -22 | -70 | 44 | 4.86 f |  |  |  |  |
| Paracentral Lobule |  |  |  |  |  |  |  |  | -20 | -46 | 50 | 5.12 |  |  |  |  |
|  |  |  |  |  |  |  |  |  | 44 | -30 | 34 | 3.57 |  |  |  |  |
| Postcentral Gyrus |  |  |  |  | 48 | -34 | 36 | 3.38 | 10 | -54 | 68 | 3.22 a |  |  |  |  |
|  |  |  |  |  | -48 | -22 | 58 | 4.48 | 36 | -38 | 72 | 4.17 |  |  |  |  |
|  |  |  |  |  | -38 | -30 | 56 | 3.63 | -24 | -44 | 68 | 5.68 |  |  |  |  |
|  |  |  |  |  | -52 | -24 | 30 | 5.05 | -38 | -24 | 42 | 4.92 df |  |  |  |  |
|  |  |  |  |  | -32 | -36 | 40 | 3.65 d | 34 | -44 | 66 | 4.78 f |  |  |  |  |
|  |  |  |  |  |  |  |  |  | 44 | -26 | 36 | 4.11 f |  |  |  |  |
| Precentral Gyrus | -60 | 2 | 32 | 3.52 | -32 | -10 | 54 | 4.36 | -26 | -28 | 66 | 4.86 |  |  |  |  |
|  |  |  |  |  | 44 | -2 | 48 | 4.42 | 40 | -8 | 46 | 4.44 |  |  |  |  |
|  |  |  |  |  |  |  |  |  | -46 | -2 | 28 | 3.69 f |  |  |  |  |

SUPPLEMENTARY TABLE S2. POST-PROCEDURAL TRAINING ACTIVITY – CONTINUED (3/4)

| Precuneus |  |  |  |  | 16 | -52 | 54 | 4.50 d | -8 | -64 | 64 | 3.32 |  |  |  |  |
| --- | --- | --- | --- | --- | --- | --- | --- | --- | --- | --- | --- | --- | --- | --- | --- | --- |
|  |  |  |  |  | 22 | -74 | 34 | 3.40 | 20 | -52 | 56 | 5.39 df | 22 | -48 | 42 | 3.95 |
| Inferior Frontal Gyrus |  |  |  |  | 54 | 10 | 16 | 3.98 | 36 | 4 | 30 | 4.56 |  |  |  |  |
|  |  |  |  |  | 44 | 4 | 28 | 4.59 | 44 | 2 | 28 | 4.38 f |  |  |  |  |
|  |  |  |  |  | 34 | 28 | 4 | 3.74 |  |  |  |  |  |  |  |  |
| Middle Frontal Gyrus | 50 | 4 | 46 | 4.34 | 44 | 8 | 58 | 4.19 d | 40 | -8 | 46 | 4.44 a |  |  |  |  |
|  |  |  |  |  | 48 | 12 | 44 | 4.11 | 16 | -8 | 62 | 4.00 |  |  |  |  |
|  |  |  |  |  | -52 | 8 | 46 | 4.69 | 52 | 14 | 46 | 3.62 |  |  |  |  |
|  |  |  |  |  |  |  |  |  | -46 | 6 | 50 | 3.25 |  |  |  |  |
|  |  |  |  |  |  |  |  |  | 42 | 8 | 58 | 3.69 df |  |  |  |  |
| Medial Frontal Gyrus |  |  |  |  | 10 | -8 | 66 | 3.84 | -4 | -12 | 72 | 4.44 |  |  |  |  |
|  |  |  |  |  | -2 | -12 | 64 | 3.59 | 6 | -8 | 62 | 4.12 |  |  |  |  |
| SMA |  |  |  |  | 12 | 0 | 50 | 3.31 d | 14 | -4 | 60 | 4.02 adf |  |  |  |  |
| preSMA |  |  |  |  | 10 | 14 | 56 | 3.97 d |  |  |  |  |  |  |  |  |
| Superior Frontal Gyrus |  |  |  |  | 16 | 8 | 70 | 4.26 | -14 | 10 | 56 | 3.56 |  |  |  |  |
| Insula | 34 | 22 | 2 | 5.43 |  |  |  |  | -32 | 26 | 20 | 3.56 |  |  |  |  |
|  | -32 | 16 | 12 | 3.64 |  |  |  |  |  |  |  |  |  |  |  |  |
| Thalamus | 16 | -16 | 4 | 3.84 | 8 | -12 | 12 | 3.93 | 6 | -14 | 12 | 3.35 a |  |  |  |  |
|  |  |  |  |  | -4 | -12 | 12 | 3.85 | -4 | -14 | 12 | 3.53 | -22 | -24 | 6 | 4.44 |
|  |  |  |  |  |  |  |  |  | -14 | -8 | 14 | 3.75 f |  |  |  |  |
| Brainstem | 6 | -22 | -12 | 6.90 | -4 | -36 | -50 | 3.73 | -6 | -20 | -24 | 3.48 | 8 | -22 | -12 | 5.45 |
|  | -4 | -22 | -14 | 5.76 |  |  |  |  | 12 | -30 | -36 | 3.47 |  |  |  |  |
| Lateral Geniculum Body |  |  |  |  |  |  |  |  |  |  |  |  |  |  |  |  |
| Caudate Nucleus |  |  |  |  | -16 | 0 | 16 | 4.30 d | -14 | -12 | 24 | 5.66 |  |  |  |  |

SUPPLEMENTARY TABLE S2. POST-PROCEDURAL TRAINING ACTIVITY – CONTINUED (4/4)

|  |  |  |  |  | -14 | -4 | 20 | 5.64 | 18 | -6 | 18 | 4.35 |  |  |  |  |
| --- | --- | --- | --- | --- | --- | --- | --- | --- | --- | --- | --- | --- | --- | --- | --- | --- |
|  |  |  |  |  | 18 | 6 | 24 | 4.05 | -18 | -6 | 16 | 5.29 d |  |  |  |  |
| Putamen |  |  |  |  | 26 | -6 | 12 | 4.07 | 20 | 4 | 16 | 4.84 |  |  |  |  |
|  |  |  |  |  | 22 | 0 | -6 | 3.51 | -26 | -8 | 10 | 4.33 f |  |  |  |  |
|  |  |  |  |  |  |  |  |  | 26 | -6 | 12 | 4.13 |  |  |  |  |
| Globus Pallidus | -12 | -4 | -4 | 4.82 | 14 | -2 | 2 | 4.64 b | -22 | -8 | -4 | 3.86 |  |  |  |  |
| Cerebellum - fastigial nucleus | -2 | -58 | -34 | 3.29 c |  |  |  |  |  |  |  |  |  |  |  |  |
| Cerebellum - Crus I |  |  |  |  |  |  |  |  | 46 | -54 | -30 | 3.21 f |  |  |  |  |
|  |  |  |  |  |  |  |  |  | 44 | -64 | -26 | 4.10 f |  |  |  |  |
| Cerebellum - Crus II |  |  |  |  |  |  |  |  | 48 | -52 | -42 | 4.16 f | -44 | -52 | -42 | 4.05 e |
|  |  |  |  |  |  |  |  |  | 46 | -52 | -44 | 4.05 |  |  |  |  |
|  |  |  |  |  |  |  |  |  | -4 | -80 | -32 | 4.36 |  |  |  |  |
| Cerebellum – area V |  |  |  |  | 20 | -54 | -14 | 3.58 | -4 | -60 | -10 | 4.17 | 32 | -36 | -36 | 3.73 |
| Cerebellum – area VI |  |  |  |  |  |  |  |  | 26 | -58 | -40 | 4.03 | -22 | -58 | -38 | 4.67 |
|  |  |  |  |  |  |  |  |  | -16 | -64 | -20 | 3.44 | -34 | -48 | -30 | 4.61 |
|  |  |  |  |  |  |  |  |  | 36 | -38 | -34 | 3.26 a |  |  |  |  |
|  |  |  |  |  |  |  |  |  | -38 | -68 | -24 | 3.28 |  |  |  |  |
| Cerebellum – area VIIB |  |  |  |  | 10 | -70 | -34 | 3.25 |  |  |  |  | 4 | -58 | -38 | 3.40 c |
| Cerebellum – area VIIIA | 8 | -62 | -40 | 3.44 |  |  |  |  | -4 | -72 | -46 | 4.71 |  |  |  |  |

a : area activated in the [session II versus I] by learning [spatial versus procedural] showing immediate post-spatial learning activity increase (Table 1a)

b : area activated in the [session III versus II] by learning [spatial versus procedural] showing delayed post-spatial learning activity increase (Table 1c)

c : area activated in the [session II versus I] by learning [procedural versus spatial] showing immediate post-procedural learning activity increase (Table 2a)

d : area activated in the [session III versus II] by learning [procedural versus spatial] showing delayed post-procedural learning activity increase (Table 2c)

e : area activated in the [session II versus III] by learning [procedural versus spatial] showing delayed post-procedural learning activity decrease (Table 2d)

f : area activated in the [session I versus II] by learning [procedural versus spatial] showing immediate post-procedural learning activity decrease (Table 2b)

g : area activated in the [session II versus III] by learning [spatial versus procedura] showing delayed post-spatial learning activity decrease (Table 1d)
